# Probiotic strain *Bacillus subtilis* CU1 stimulates immune system of elderly during common infectious disease period: a randomized, double-blind placebo-controlled study

**DOI:** 10.1186/s12979-015-0051-y

**Published:** 2015-12-03

**Authors:** Marie Lefevre, Silvia M. Racedo, Gabrielle Ripert, Béatrice Housez, Murielle Cazaubiel, Corinne Maudet, Peter Jüsten, Philippe Marteau, Maria C. Urdaci

**Affiliations:** Lesaffre Human Care, Lesaffre Group, 278 Avenue de la Marne, Château rouge, 59700 Marcq en Baroeul, France; University of Bordeaux, UMR 5248, Bordeaux Sci Agro, Gradignan, France; Biofortis-Mérieux NutriSciences, Saint-Herblain, France; Paris 7 University and AP-HP, Hôpital Saint Antoine, Paris, France

**Keywords:** Clinical trial, Elderly, Common infectious disease, Probiotics, Immunostimulation

## Abstract

**Background:**

*Bacillus* probiotics health benefits have been until now quite poorly studied in the elderly population. This study aimed to assess the effects of *Bacillus subtilis* CU1 consumption on immune stimulation and resistance to common infectious disease (CID) episodes in healthy free-living seniors.

**Results:**

One hundred subjects aged 60–74 were included in this randomized, double-blind, placebo-controlled, parallel-arms study. Subjects consumed either the placebo or the probiotic (2.10^9^*B. subtilis* CU1 spores daily) by short periodical courses of 10 days intermittently, alternating 18-day course of break. This scheme was repeated 4 times during the study. Symptoms of gastrointestinal and upper/lower respiratory tract infections were recorded daily by the subjects throughout the study (4 months). Blood, saliva and stool samples were collected in a predefined subset of the first forty-four subjects enrolled in the study. *B. subtilis* CU1 supplementation did not statistically significantly decrease the mean number of days of reported CID symptoms over the 4-month of study (probiotic group: 5.1 (7.0) d, placebo group: 6.6 (7.3) d, *P* = 0.2015). However, in the subset of forty-four randomized subjects providing biological samples, we showed that consumption of *B. subtilis* CU1 significantly increased fecal and salivary secretory IgA concentrations compared to the placebo. A post-hoc analysis on this subset showed a decreased frequency of respiratory infections in the probiotc group compared to the placebo group.

**Conclusion:**

Taken together, our study provides evidence that *B. subtilis* CU1 supplementation during the winter period may be a safe effective way to stimulate immune responses in elderly subjects.

## Background

Viral respiratory and gastrointestinal infections are a predominant cause of morbidity and mortality in the elderly whose ageing immune system contributes significantly to poor outcomes [1]. Ageing is associated with a decline of innate and adaptive immune responses. For innate dysfunction, it has been described that the function of natural killer cells, dendritic cells [2], macrophages [3] and neutrophils [4] decrease in the elderly. Moreover, age-dependent thymic involution leads to the reduction of circulating naive T cells and the increase frequency of regulatory, memory and effector T cells [5, 6]. A dramatic reduction in B cell repertoire associated with a decreased systemic antibody response to vaccination has been observed in the elderly population [7] showing that the B cell compartment is also affected by ageing [8, 9]. In addition, the production of secretory IgA (SIgA) at the mucosal surfaces decreases with age and can lead to an increased risk of infection [10, 11]. SIgA, the predominant immunoglobulin class in human external secretions, is a key element in the maintenance of gut microbiota homeostasis and in the protection of gastrointestinal and respiratory tracts against pathogens [12]. Moreover, it has been also shown that the age-dependent modifications of the composition of the gut microbiota also contribute to the defective local and systemic immune defenses in the elderly population [13, 14].

The development of strategies aimed at counterbalancing the immune frailty in the elderly is a major challenge for 21^st^ century medicine. Nutritional supplementation, including probiotics, in this population may help maintain immune function either by direct interaction with the host immune system or indirectly by re-equilibrating the gut microbiota [15–17].

Probiotics have been defined as ‘Live microorganisms which when administered in adequate amounts confer a health benefit on the host’ [18]. *Lactobacillus* and *Bifidobacterium* are the most commonly used bacterial probiotics. Fermented milk or dairy products containing *Lactobacillus* have shown effects on duration or frequency of respiratory and gastrointestinal infections [19–21] and reduced the risk of the common cold in healthy elderly subjects [22]. Mañé et al. [23] showed significant trends in reducing infection incidence and mortality due to pneumonia in institutionalized elderly subjects treated with two *Lactobacillus plantarum* strains. Some trials showed that *Lactobacillus* and *Bifidobacterium* probiotics could increase influenza vaccination immune responses in the elderly [24–27].

Endospore formers such as *Bacillus* species are interesting because their spores resist the acid barrier of the stomach and are stable for long periods in commercial food products [28]. Bacilli, considered as gut commensals, have been used as probiotics for prophylaxis of human gastrointestinal disorders, to prevent recurrent respiratory infections or as an adjunct to antibiotic use [29–34].

Probiotics have been suggested to protect against infectious diseases by several strain-dependant mechanisms [35, 36] including secretion of anti-pathogen substances, competitive exclusion of pathogens, maintenance of mucosal integrity and stimulation of systemic or mucosal immune responses [35–39]. *Bacillus* species have been shown to produce antimicrobial substances [40, 41], to enhance epithelial gut barrier functions [42, 43] and stimulate cytokine [43–45] and SIgA in humans [46].

In this study we evaluated the beneficial effect of *Bacillus subtilis* CU1 administration in an elderly population. This probiotic displays immunostimulating properties and antagonizes gastrointestinal pathogen infection by producing antimicrobial substances such as amicoumacins (Racedo SM & Urdaci MC, unpublished observations). This randomized, double-blind placebo-controlled study investigated the effect of probiotic strain *B. subtilis* CU1 intake on resistance to common infectious disease (CID), notably by measuring mean cumulative number of days with CID in healthy free-living seniors (individuals over 60 years old). As secondary endpoints, the study examined the effect of *B. subtilis* CU1 intake on the stimulation of the mucosal and systemic immune response by measuring intestinal and salivary SIgA levels and serum cytokine levels, respectively, in a subset of 44 subjects.

## Results

### Subject characteristics

**Fig. 1:**
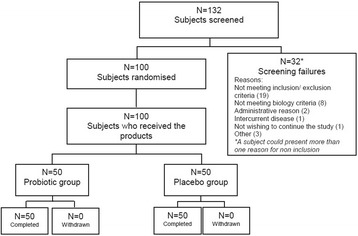
Flow chart of subjects

One hundred thirty two (132) subjects were screened for study eligibility, and 100 were randomized to the probiotic group (*N* = 50) or to the placebo group (*N* = 50) (Fig. 1). All enrolled subjects completed the study without major deviation.

The baseline characteristics for the population are presented in Table 1. The data from the initial clinical examination were normal for all volunteers. Mean age observed in the probiotic group (63.3 (2.8) years of age) and placebo group (63.0 (2.4) years of age) were consistent with inclusion criteria. The influenza vaccination rates in the subjects, seasonal influenza and influenza A, from the beginning of the influenza vaccination season (September) were respectively 16. 0 % and 8.0 % in probiotic group and 14.0 % and 12.0 % in placebo group. A good mean compliance was observed (>99 % in both groups).

**Table 1:** Baseline subject characteristics of the whole population (*N* = 100), by product group

	Probiotic group		Placebo group	
	(*N* = 50)		(*N* = 50)	
	Mean	SD	Mean	SD
Age (years)	63.3	2.8	63.0	2.4
Body weight (kg)	60.5	22.5	57.4	21.2
Body mass index (kg/m^2^)	25.5	5.0	24.9	4.0
Females				
*n*	40		39	
%	80.0		78.0	
CID during previous winter	2.7	1.0	3.2	1.2
Vaccination				
Seasonal *influenza*				
*n*	8		7	
%	16.0		14.0	
*Influenza A*				
*n*	4		6	
%	8.0		12.0	
*Pneumococcus*				
*n*	0		0	
%	0.0		0.0	

Mean values and standard deviations; numbers and percentages

### Clinical outcomes on the whole study population

Considering the whole study population (*N* = 100), the mean number of days with CID symptoms over the 4-month study period was 5.1 (7.0) d in the probiotic group and 6.6 (7.3) d in the placebo group (*P* = 0.2015) (Table 2). The percentage of subjects reporting at least one CID episode during the study was 58.0 % in the probiotic group (*N* = 29/50) and 66.0 % in the placebo group (*N* = 33/50) (*P* = 0.4106). The risk to report an infectious episode in the probiotic group was 12 % lower than in the placebo group (*Relative Risk* = 0.88 [1.20;0.65]). There was no statistically significant difference between the probiotic and the placebo groups in mean duration, intensity, and frequency of CID during the observation period (*P* = 0.2361, *P* = 0.7400, and *P* = 0.3290 respectively).

**Table 2:** Effect of probiotic (*B. subtilis* CU1) and placebo consumption on clinical outcomes of infectious diseases

	Whole population (*N* = 100)					Subset of population (*N* = 44)				
	Probiotic group (*N* = 50)		Placebo group (*N* = 50)		*P*	Probiotic group (*N* = 22)		Placebo group (*N* = 22)		*P*
	Mean	SD	Mean	SD		Mean	SD	Mean	SD	
Mean number of days with CID	5.1	7.0	6.6	7.3	0.2015^a^	4.5	7.3	7.3	8.2	0.1101^a^
Mean duration of CID (d)	5.0	4.6	5.3	4.1	0.2361^a^	5.8	5.6	5.7	4.1	0.2361^a^
Mean intensity of CID	8.1	5.0	7.6	4.4	0.7400^a^	9.0	6.2	8.8	5.3	0.7400^a^
CID frequency	1.0	1.2	1.2	1.2	0.3290^a^	0.8	1.0	1.3	1.2	0.1117^a^
Subjects with at least one CID					0.4106^b^					0.1260^b^
*n*	29		33			11		16		
%	58 · 0		66 · 0			50 · 0		72 · 7		
Mean number of days with RI	4.4	6.9	6.2	7.2	0.1027^a^	3.7	6.9	6.6	7.9	0.0818^a^
Mean duration of RI (d)	5.9	5.0	5.6	4.2	0.9043^a^	6.8	6.3	6.1	4.3	0.9325^a^
Mean intensity of RI	9.3	5.3	7.8	4.6	0.1428^a^	11.1	6.3	9.3	5.6	0.3473^a^
RI frequency	0.7	0.9	1.1	1.2	0.1181^a^	0.6	0.7	1.1	0.9	**0.0323** ^a^
Subjects with at least one RI					0.1609^b^					0.0701^b^
*n*	24		31			10		16		
%	48.0		62.0			45.5		72.7		

Data are presented for the whole population (*N* = 100) and the subset of population with biology analysis (*N* = 44). (Mean values and standard deviations; numbers and percentages)

Statistical differences were evaluated using Wilcoxon-Mann-Whitney’s test or Savage’s test according to the asymmetry of data (^a^), or logistic regression model (^b^)

### Clinical outcomes on the subset of 44 subjects (post-hoc analysis)

Considering the subset of the 44 subjects, the mean number of days with CID symptoms was 4.5 (7.3) days in the probiotic group and 7.3 (8.2) days in the placebo group (*P* = 0.1101) (Table 2). In this subset, the percentage of subjects reporting at least one infectious episode during the study was 50.0 % (*N* = 11/22) in the probiotic group and 72.7 % (*N* = 16/22) in the placebo group (*P* = 0.1260). The risk to report an infectious episode was 31 % lower in probiotic group than in placebo group (*Relative Risk* = 0.69 [1.12;0.42]). There was no difference in mean duration, intensity and frequency of CID during the observation period between the probiotic and placebo groups (*P* = 0.2361, *P* = 0.7400, and *P* = 0.1117 respectively). In the same subset (*N* = 44), the frequency of respiratory infections was significantly lower in the probiotic group compared to the placebo group (*P* = 0.0323): a mean number of 0.6 (0.7) respiratory infections was observed in the probiotic group vs. 1.1 (0.9) in the placebo group. The mean number of days with respiratory CID symptoms was 3.7 (6.9) in the probiotic group and 6.6 (7.9) in the placebo group (*P* = 0.0818).

### Immunological parameters (subset of 44 subjects)

#### Mucosal response

**Fig. 2:**
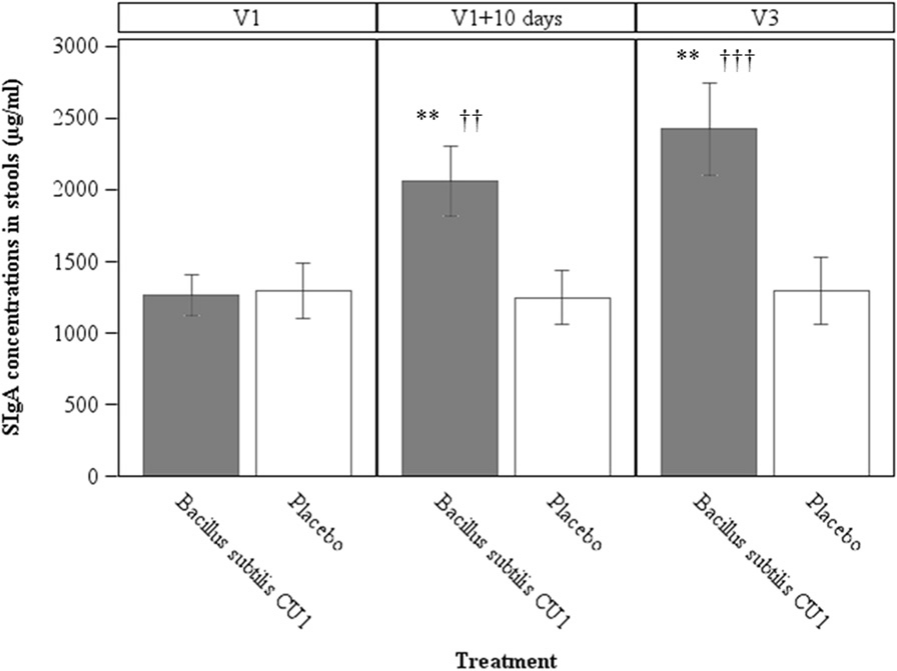
Concentrations of secretory IgA in stools. Fecal SIgA concentrations were assessed in subjects from the subset of population (*N* = 44), at baseline (V1), after 10 days of consumption of study products (V1 + 10 d) and at the end of the study (V3). Values are means, with standard error of means represented by vertical bars. Fecal SIgA concentrations were significantly higher in the probiotic group compared to the placebo group (**P <0.01), and significantly increased in the probiotic group during the study (†† *P* < 0.01, ††† *P* < 0.001)

**Fig. 3:**
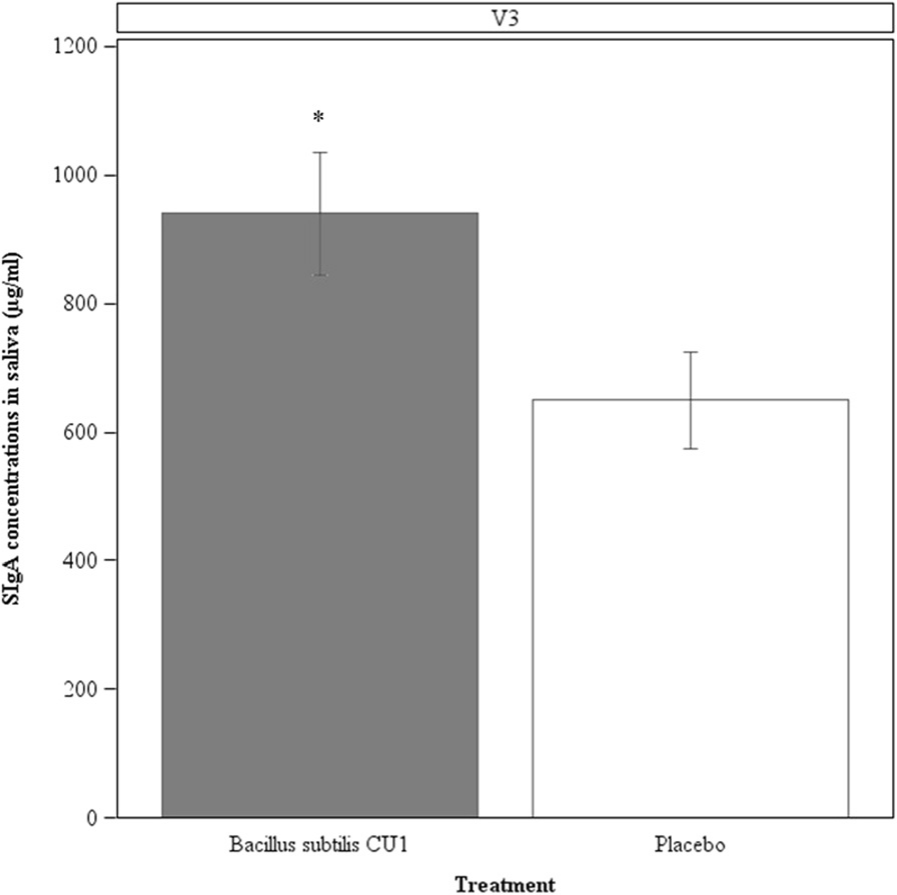
Concentrations of secretory IgA in saliva. Salivary SIgA concentrations were assessed in subjects from the subset of population (*N* = 44), at the end of the study (V3). Values are means, with standard error of means represented by vertical bars. Salivary SIgA concentration was significantly higher in the probiotic group compared to the placebo group (*P <0.05)

*Fecal and salivary SIgA response*. SIgA is widely used as marker of mucosal immunity in clinical studies [47]. Remarkably, we observed a significantly higher concentration of SIgA in stools in the probiotic group compared to the placebo group after 10 d of product consumption (probiotic group: 2062.6 (1161.8) μg/ml; placebo group: 1249.5 (863.8) μg/ml; *P* = 0.0038) (Fig. 2). The increased SIgA levels were still observed at the end of consumption and 18 d after the end of *B. subtilis* CU1 consumption (probiotic group: 2424.4 (1252.3) μg/ml; placebo group: 1297.1 (953.7) μg/ml; *P* = 0.0032). Furthermore, fecal SIgA concentrations significantly increased between pre- and post-supplementation with *B. subtilis* CU1 after 10 days of probiotic consumption (*P* = 0.0012). Elevated levels persisted 18 d after the last probiotic consumption (*P* = 0.0008). SIgA concentrations in stools were not statistically affected by placebo consumption. In addition, a significantly higher concentrations of salivary SIgA were observed in the probiotic group compared to the placebo group at the end of consumption and 18 d after the end of *B. subtilis* CU1 consumption (probiotic group: 940.4 (446.0) μg/mL; placebo group: 650.1 (343.5) μg/mL; *P* = 0.0219) (Fig. 3).

#### Systemic response

**Fig. 4:**
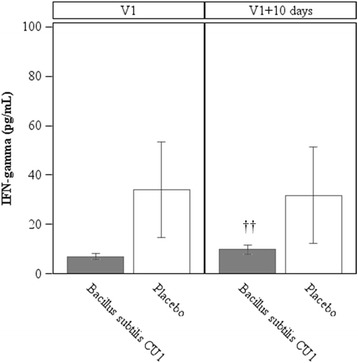
Concentrations of IFN-gamma in blood. IFN-gamma concentrations were assessed in subjects from the subset of population (*N* = 44), at baseline (V1), after 10 days of consumption of study products (V1+ 10 d) and at the end of the study (V3). Values are means, with standard error of means represented by vertical bars. IFN-gamma concentrations were significantly increased in the probiotic group between V1 and V1 + 10 d (††*P* <0.01)

*Blood IFN response* (Fig. 4)*.* No statistically significant difference in IFN-gamma concentrations were observed between groups, after 10 d of probiotic consumption (V1 + 10 d) (probiotic group: 9.7 (8.1) pg/mL; placebo group: 31.8 (92.4) pg/mL; *P* = 0.0981). However, IFN-gamma concentrations significantly increased in the probiotic group from first pre- to post- supplementation, after 10 d of probiotic consumption (probiotic group at V1: 6.9 (5.0) pg/mL; *P* = 0.0090), while no significant change was observed in the placebo group. No statistically significant differences in the plasma concentrations of cytokines (IL-1beta, IL-4, IL-6, IL-8, IL-10, IL-12p70, IgA, and TNF-alpha) were measured between the probiotic and the placebo groups from pre- to post- supplementation.

### Numeration of *B. subtilis *in the stools (subset of 44 subjects)

An increase in *Bacillus* spore concentrations were observed in stools of subjects from the probiotic group but not in the placebo group (Table 3). Molecular typing using the OPL12 primer showed the presence of the *B. subtilis* CU1 strain in stools as opposed to the placebo group and demonstrates the viability of the probiotic strain.

**Table 3:** Bacterial counts of *Bacillus* spores in the stools of the subset population (*N* = 44)

	Whole population (*N* = 100)			
	Probiotic group (*N* = 22)		Placebo group (*N* = 22)	
	Mean	SD	Mean	SD
V1	2.5.10^3^	3.0.10^3^	2.5.10^3^	5.4.10^3^
V1 + 10 days	1.9.10^7^	1.1.10^7^	2.3.10^3^	5.1.10^3^
V3	7.5.10^3^	1.5.10^4^	3.0.10^3^	4.4.10^3^

Mean values and standard deviations

### Stool cytotoxicity (subset of 44 subjects)

No statistically significant difference was measured in the cytotoxicity levels in stools between the probiotic group (94.25 % (34.46) of viability) and placebo group (93.98 % (31.06) of viability), after 10 d of product consumption (*P* = 0.6328). These data confirm the absence of stool cytotoxicity induced by *B. subtilis* CU1.

### Safety (whole study population)

Investigators reported 108 adverse events in the probiotic group and 85 in the placebo group (*P* = 0.8369). There were no abnormal values of biological parameters at the end of the study, and no clinically significant variation was observed during the study, on renal and hepatic functions.

## Discussion

The current controlled study was designed to evaluate the effect of probiotic strain *B. subtilis* CU1 consumption (2.10^9^spores/d) on immune system stimulation and resistance to respiratory and gastrointestinal CID in healthy free-living seniors with known past histories of CID during the winter period. The demographic characteristic of the volunteers, the duration of study and the compliance to product consumption were similar for probiotic and placebo groups. The probiotic product was safe and well tolerated. An increase in *B. subtilis* CU1 concentration was observed in stools after intake of the probiotic product, which suggests survival of the strain in the gastrointestinal tract and is consistent with high compliance of study assessed by product consumption. The probiotic did not significantly affect CID in the whole population (*N* = 100). In the subset of 44 subjects, and as planned in the protocol, biological explorations were performed. These analyses showed that *B. subtilis* CU1 significantly increased the levels of SIgA in stools (*P* = 0.0032) and saliva (*P* = 0.0219) in comparison to placebo and induced significantly higher levels of serum IFN-gamma (*P* = 0.0090). Furthermore, a post-hoc analysis in this subset of subjects showed a statistically lower frequency of respiratory infections in the probiotic group compared to the placebo group (*P* = 0.0323).

We readily acknowledge several limitations to our study. Except for respiratory infections in the subset of 44 subjects, the clinical efficacy of oral administration of probiotic *B. subtilis* CU1 did not reach statistical significance. Consequently the study hypothesis was not reached, i.e., to observe a difference of 3 d in CID episodes between the two groups. However the statistical power of our study was lower than expected and *B. subtilis* supplementation tended to decrease the mean number of days with respiratory CID symptoms compared to the placebo group in the subset of 44 subjects (post-hoc analysis). In addition, *B. subtilis* CU1 supplementation significantly reduced the frequency of respiratory infections in this subset of population (*P* = 0.0323). This subgroup was originally planned in the protocol only for biological analysis and the clinical efficacy observed in this subset, while not confirmed in the whole population, might be explained either by chance only or by the higher infection rates at the beginning of the clinical study (i.e., beginning of winter season, which is likely to correspond to the highest CID exposure period).

### B. subtilis CU1 stimulates systemic immune response

In the present study, we observed that supplementation with *B. subtilis* CU1 stimulated systemic immune response in seniors by significantly increasing serum IFN-gamma in the probiotic group following first *B. subtilis* supplementation. Concentrations of other measured serum cytokines and serum IgA were not significantly modified. These results are in accordance with previous mouse studies using *B. subtilis* CU1 (Racedo SM & Urdaci MC, unpublished observations). Huang et al. [48] also found that *Bacillus* strains could stimulate systemic and intestinal IFN-gamma production in mice. This Pro-Th1 cytokine plays a role in the host defense against several infectious diseases, including viral infection and has a variety of immune functions such as stimulation of macrophages and natural killer cells [49, 50]. Different studies have emphasized the importance of IFN-gamma production for the protective effect of probiotics against influenza infection [51, 52]. Further investigations in *B. subtilis* CU1’s capability in increasing serum IFN-gamma levels and the strengthening of the systemic anti-viral and anti-bacterial immune defenses in the elderly population would be interesting.

### B. subtilis CU1 enhances intestinal and respiratory mucosal immune responses

An important finding of the present study was that, compared to placebo, the oral intake of *B. subtilis* CU1 resulted in higher SIgA production in the healthy seniors. Ten days of probiotic intake were sufficient to increase stool SIgA levels 65 % in treated seniors compared to placebo. Moreover, an increased level of 87 % was maintained at least 18 days after the last probiotic administration. Previous controlled clinical trials have shown that the intake of probiotic bacteria (mainly *Lactobacillus* and *Bifidobacterium*) stimulates mucosal immune systems by enhancing fecal IgA [53, 54]. Kabeerdoss et al. [54] observed an increase of fecal IgA during probiotic intake with a subsequent decrease after cessation of administration of the probiotic.

We assessed SIgA concentrations in saliva at the end of the 18-d follow up period. *B. subtilis* CU1 intake produced a 45 % increase in SIgA relative to placebo group. Since *B. subtilis* CU1 had been shown to increase IgA producing B cells in Peyer’s patches in mice (Racedo SM & Urdaci MC, unpublished observations), one can postulate that *B. subtilis* CU1 consumption strengthens the generation of α4β7^+^IgA^+^ B cells in the Peyer’s patches of the small intestine of elderly subjects. The homing of these IgA producing B cells to the intestinal mucosa and the salivary glands [55] most probably accounts for the high SIgA levels measured in the feces and saliva of the probiotic group. In the elderly, only one previous study has shown that oral administration of heat-killed *Lactobacillus pentosus b240* produced an increase in salivary IgA secretion [16]. Importantly, it has been shown, in mice, that 50 % increase production of intestinal SIgA to a bacterial toxin significantly increased the vaccine-induced protection directed against the toxin [56]. Additionally, it has also been shown that a 20 % increase in production of total SIgA in saliva is associated with a decrease in the incidence of colds and flu-like symptoms in humans [57, 58]. Therefore, increased SIgA levels of 87 % and 45 % in faeces and saliva respectively are most probably of physiological significance in ameliorating the health status of seniors receiving *B. subtilis* CU1.

Taken together, these findings show the utility of oral administration of probiotic *B. subtilis* CU1 to increase mucosal immune responses. The increased SIgA levels in the intestine and saliva might contribute to strengthening the mucosal anti-viral and anti-bacterial immune defenses of the elderly population. It has to be noted that secretion of salivary SIgA has been shown to be impaired by stress such as academic stress or intensive physical exercise [59, 60]. Therefore interesting future work would be to investigate whether *B. subtilis* CU1 stimulation of mucosal immune system might be beneficial in the general population, notably in a population under stress.

King et al. [61] recently published a meta-analysis and systematic review showing that *Lactobacillus spp.* and *Bifidobacterium spp.* strains brought by food products or supplements significantly lowered the number of days of acute respiratory infections in a healthy population of children and adults and shortened acute respiratory infectious periods. There are only few studies in the elderly and the present one is the first to indicate a trend toward a reduction of CID by a *B. subtilis* strain in this population. Additional larger clinical trials have to be conducted to confirm these clinical outcomes.

## Conclusions

Despite the absence of significant results on CID in the whole population, the present study showed that consumption of *B. subtilis* CU1 significantly increased intestinal and salivary SIgA and serum IFN-gamma levels in a subset senior population. It suggests that daily *B. subtilis* CU1 supplementation during the winter months may be a safe effective way to stimulate systemic and mucosal immune responses of the elderly. However, no firm conclusion can be made about the effect of *B. subtilis* CU1 supplementation on CID. Additional larger clinical trials have to be conducted to confirm these clinical outcomes.

## Methods

### Ethics, consent and permissions

The study was approved by the West IV Ethics Committee for Human Research and the French Health Products Safety Agency (AFSSAPS), France and was performed in accordance with the principles of the Declaration of Helsinski, of Good Clinical Practice (Directive ICH E-6, 24 November 2006), and current French regulations (Code de Santé Publique, Titre II du livre Premier). All participants had given their written informed consent before inclusion in the study.

### Study subjects

One hundred (100) healthy free-living adults between 60 and 74 years of age, without any known congenital or immune defects, severe chronic disease or allergies and reporting at least two CID episodes during the previous winter period were recruited. Subjects were not included if any of the following criteria applied: chronic respiratory insufficiency, cardiac insufficiency, cancer (chemotherapy, radiotherapy), unstabilized chronic disease (severe renal or hepatic insufficiency, etc.), or any chronic severe affection likely to interfere with evaluation of the study parameters. For the inclusion, subjects were not allowed to take any drug potentially known to interfere with the evaluation of the study parameters, including corticoids and immunosuppressive drugs. Consumption of dietary supplements was forbidden in the last 2 months before inclusion, as well as regular consumption of probiotic products 1 month before the start of the study.

### Study design

The study was a monocentric, randomized, double-blind, placebo-controlled, parallel arms trial. Subjects were randomly allocated to the probiotic group or to the control group.

The study lasted 16 weeks and consisted of four consumption periods of 10 d each, followed by 18 d without consumption of the study products (break period).

**Fig. 5:**
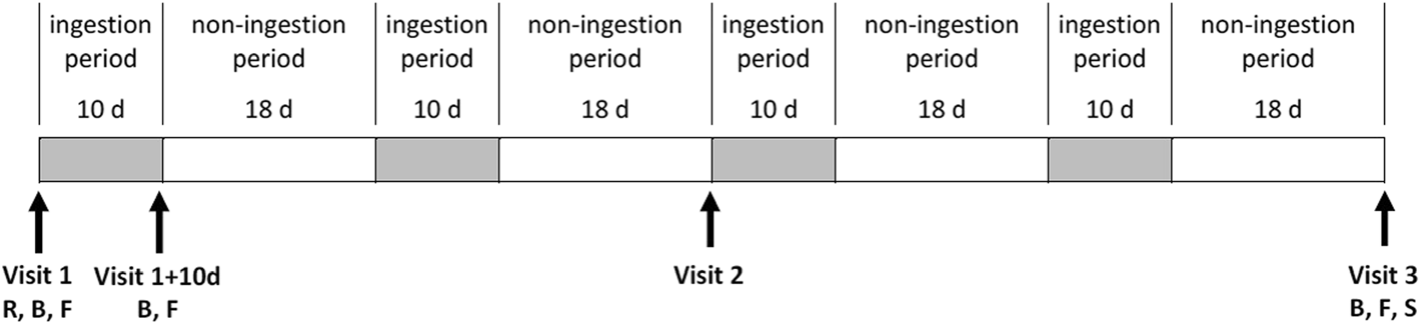
Study design. R indicates randomization of the 100 subjects. Blood samples (B), fecal samples (F) and salivary samples (S) concerned a subset of 44 subjects

At the selection visit, 1 or 2 weeks before initiation of the study, volunteers underwent a clinical examination and blood was taken from volunteers to assess safety parameters and measure of biological inclusion parameters. The investigators checked for inclusion criteria and recorded the subject’s demographic characteristics. Eligible volunteers were randomly allocated by the investigator to the probiotic or the placebo group on Visit 1 (V1) of the study, according to the randomization list. Randomization was done without stratification using SAS® software version 9.1.3 Service Pack 4 (SAS Institute Inc., Cary, NC, USA). The randomization list was prepared before the beginning of the study by a person not related to the clinical phase, the data management or statistics. In addition, it was prepared and stored confidentially. The unblinding envelopes were concealed from the person responsible for randomization. During the study, four visits were planned: V1 (inclusion visit, beginning of the 1^st^ product consumption period), V1 + 10 d (follow-up visit, 10 days after V1, end of the 1^st^ product consumption period), V2 (follow-up visit, 2 months after V1) and V3 (end-of-study visit, 4 months after V1) (Fig. 5).

The trial was conducted during the winter season 2010–2011, in the Nantes area, France.

### Study products

Study products were presented in the form of food supplements. The probiotic product consisted of *B. subtilis* CU1 mixed with excipients (maltodextrin DE14, dicalcic phosphate, magnesium stearate, colloidal silica). Each probiotic capsule contained 2.10^9^ spores of *B. subtilis* CU1 (LESAFFRE, Marcq en Baroeul, France). The probiotic preparation contains 95 % of *B. subtilis* spores and 5 % of vegetative cells. Due to spore stability over time, the probiotic counts were the same at beginning and at the end of study. The placebo capsule contained only the excipient mix. Placebo products were indistinguishable from the probiotic product in appearance, smell and taste.

During the four 10-d product consumption periods, the subjects were instructed to consume daily one capsule of the study product, in the morning (40 min before breakfast). Otherwise, subjects were asked not to modify their food habits and they were prohibited from taking any dietary supplement or food product containing probiotics during the study.

### Study endpoints

The primary endpoint was a between products comparison of the mean cumulative number of days with CID (upper and lower respiratory tract infections, gastrointestinal tract infections) during the 4 months of study in the whole population (*N* = 100). In addition, some clinical endpoints were analysed in the whole population, and biological endpoints in the subset of the first 44 subjects (half in the *B. subtilis* group, half in the placebo group).

Clinical endpoints included the mean duration of CID, the intensity of CID, the frequency of CID, and the percentage of subjects with at least one CID (*N* = 100). Biological endpoints included blood immunological marker concentrations (IL-1beta, IL-4, IL-6, IL-8, IL-10, IL-12p70, TNF-alpha, IgA, IFN-gamma, at V1 and V1 + 10 d), salivary secretory IgA concentration (at V3), and fecal secretory IgA concentration (at V1, V1 + 10 d and V3) (*N* = 44). At last, the presence of *B. subtilis* in stools and the cytotoxicity of stools were analysed in the subset population (*N* = 44).

### Common infectious disease symptoms

During the 4 months of study, subjects were instructed to track any CID symptoms they had in a diary. The items detailed in this diary were: symptoms of gastrointestinal and upper/lower respiratory tract infections (cough, hoarseness, sore throat, itchy throat, rhinorrhea, sneezing, nasal obstruction, conjunctivitis, fatigue, headache, myalgia, nausea), body temperature, vomiting, and diarrhoea.

The intensity of each symptom of gastrointestinal and upper/lower respiratory tract infections was rated on a 4-point scale (from 0: no symptom to 3: severe symptom) whereas fever (i.e., body temperature increased from at least one degree higher compared to basal temperature), vomiting and diarrhoea (i.e., more than 3 liquid stools per day) were rated on dichotomous scales (0 = absence; 3 = experience of the sign). All symptoms were reviewed by the investigator who determined if they complied with the diagnosis of a CID or not. When a CID was diagnosed, a symptomatic score was calculated on a daily basis, taking into account CID symptoms, fever, vomiting, and diarrhoea. This score rates from 0 to 45 arbitrary units.

For analysis of the mean number of days with CID and the frequency of CID, all subjects were considered and “0” was applied to subjects without CID.

### Sample collection

Fasting blood samples and stool samples were collected at V1, V1 + 10 d and V3 among the first 44 randomized subjects (*N* = 22 in probiotic group, *N* = 22 in placebo group), which corresponded to the first twenty-two subjects enrolled in each group. The randomized repartition of the subjects was ensured by the randomization list. Blood was centrifuged for 10 min at 1,000 x g at 4 °C to separate serum. An aliquot of the serum was used for IgA determination and a second aliquot was collected for cytokine analysis. Additionally, saliva samples were collected at V3 in the same subset. Biological samples, serum, stools and saliva were stored frozen at −80 °C until analysis.

### Serum, stools and saliva Immunoglobulin A (IgA) quantification

Serum IgA concentration was measured by Elisa (enzyme-linked immunosorbent assay) according to the manufacturer’s instructions (Hitachi 911, Roche, Basel, Switzerland). SIgA in stools and saliva were measured by Elisa (Immuchron AG, Heppenheim, Germany) according to the manufacturer’s instructions.

### Serum cytokine quantification

Interleukins (IL-1beta, IL-6, IL-8, IL-10, IL-12p70), Tumor Necrosis Factor alpha (TNF-alpha) and Interferon gamma (IFN-gamma) were measured using Human Th1/Th2 11plex FlowCytomix immunoassays (eBioscience, Inc) on the BD Accuri™ C6 Flowcytometer (BD Bioscience) and ELISA (Ready-SET-Go® eBioscience, Inc.). Furthermore, human Interferon beta (IFN-beta) was analyzed by Elisa (R&D Systems, Inc.). All assays were performed according to the manufacturer’s instructions.

### Bacillus spores counts in stools and CU1 strain identification

Stools samples (2 g) were placed in Stomacher® 400 classic bags, diluted with 18 ml of sterile physiological saline water and homogenized for 2 min. Ten ml of dilutions were heated (80 °C for 10 min) in order to kill bacterial vegetative cells. Samples were serially diluted 1:10 in sterile saline and plated on Mueller- Hinton Agar plates (Difco BD Laboratory, Franklin Lakes, USA) for 24 h at 37 °C for spores counts.

In order to identify CU1 colonies in plates, a molecular typing method RAPD (Randomly Amplified Polymorphic DNA) was used. Different types of colonies from every fecal sample were analyzed in triplicate using the OPL12 primer that generates a specific profile for the CU1 strain [62].

### Assessment of stool cytotoxicity

Stool samples (1 g) were diluted in 5 ml of Dulbecco’s modified Eagle’s minimum essential medium (DMEM). After homogenization, samples were centrifuged at 10000 g for 10 min, then filtered through a 0.2 μm membrane filter and diluted 1/20 (volume/volume (v/v)) in DMEM media. Stool cytotoxicity was evaluated by quantifying Vero cell detachment.

Vero cells were grown in DMEM containing 25 mM glucose (Sigma-Aldrich), supplemented with 10 % (v/v) heat-inactivated fetal calf serum (Gibco), 1 % (v/v) nonessential amino acids (Sigma), penicillin (100 UI/ml), streptomycin (100 μg/ml), gentamicin (50 μg/ml) (Sigma). For maintenance purposes, cells were passed every 3 d, using Accutase Solution (Sigma). Monolayers were prepared in 48-well tissue culture plates (Greiner Bio One, Germany) by seeding 5.10^4^ cells per well. Experiments and cell maintenance were carried out at 37 °C in a 5 % CO_2_**-**95 % air atmosphere. Fully confluent cells (3–4 d in culture) were used throughout.

Briefly, confluent cells were co-incubated with the different stool samples at 37 °C for 2 h and then cells were washed with phosphate-buffered saline (PBS), fixed for 1 min with 2 % (v/v) formaldehyde in PBS, washed again in PBS and stained with 500 μl of crystal violet solution (0.13 % crystal violet, 5 % ethanol, and 2 % formaldehyde in PBS w/v/v) for 20 min at room temperature. After being exhaustively washed with PBS to remove stain excess, samples were treated for 1 h with freshly prepared 50 % ethanol in PBS (v/v) at room temperature. Absorbance was measured at 650 nm in a Thermo Max Microplate spectrophotometer reader. Percentage of attached cells was calculated as: *100 x (****A****/****Ac****)*, where **A** is the absorbance of treated cells and **Ac** is the absorbance of untreated control cells. As positive control a diluted supernatant of *Clostridium difficile* VPI 10463 was used.

### Safety

Adverse events were collected during the study by investigators and reported in the case report forms of each subject. The investigators had also to evaluate imputability of any adverse event to the study products.

### Statistical analysis

Based on previous pilot clinical studies, sample size was calculated to detect an intergroup difference of 3 d in CID episodes (with Standard Deviation (SD): 5 d), using a two-tailed *t*-test at the significance level of 0 · 05. Forty-eight subjects were required in each group to provide 80 % statistical power. To account for potential drop-outs, it was planned to include fifty subjects in each group.

Data were analysed using SAS® software version 9.1.3 Service Pack 4 (SAS Institute Inc., Cary, NC, USA). Results are expressed as Mean (SD). Significance was set at *P* < 0.05.

The mean number of days with CID and other clinical outcomes (mean duration, intensity, and frequency of CID) were compared between groups using the Wilcoxon-Mann-Whitney’s test or Savage’s test (according to the asymmetry of data). The percentage of subjects with at least one CID was compared between-group using a logistic regression model. Immunological variables with a normal distribution (plasma, salivary and fecal IgA, IL-6, IL-8, IL-10, IL-12, and stool cytotoxicity) were compared using Student’s *t*-test for between-group analysis and Student’s paired *t*-test for within-group analysis. For criteria which do not respect normal distribution (IFN-gamma, TNF-alpha, IL-1 and IL-4), a Wilcoxon-Mann-Whitney’s test was applied for between group analysis, and completed with Savage’s test for IL-1 and IL-4 due to dissymmetry of these biological parameters. Within-group analysis was performed using Wilcoxon’s paired test. The number of subjects with at least one side-effect in each group (probiotic and placebo groups) was compared using Chi-square test.

Following this primary analysis, some post-hoc analyses were performed. The first analysed the clinical outcomes reported in the subset of 44 subjects (number of days with CID, mean duration of CID, intensity of CID, frequency of CID, and percentage of subjects with at least one CID). The second post-hoc analysis applied on the clinical outcomes in subjects who reported at least one symptom of upper/lower respiratory tract infections, whatever this symptom was or not associated to a symptom of gastrointestinal infection. This latter analysis was performed in the whole population of 100 subjects and in the subset of 44 subjects. The statistical models were identical to the ones applied for the primary analysis.

## Abbreviations

CID: Common Infectious Disease
DMEM: Dulbecco’s Modified Eagle’s Minimum Essential Medium
PBS: Phosphate-Buffered Saline
SD: Standard Deviation
v/v: volume/volume
w/v/v: weight/volume/volume
